# Consenting options for posthumous organ donation: presumed consent and incentives are not favored

**DOI:** 10.1186/1472-6939-13-32

**Published:** 2012-11-22

**Authors:** Muhammad M Hammami, Hunaida M Abdulhameed, Kristine A Concepcion, Abdullah Eissa, Sumaya Hammami, Hala Amer, Abdelraheem Ahmed, Eman Al-Gaai

**Affiliations:** 1 Clinical Studies and Empirical Ethics Department, King Faisal Specialist Hospital and Research Centre, P O Box # 3354 (MBC 03), Riyadh, 11211, Saudi Arabia; 2 Alfaisal University college of Medicine, Riyadh, Saudi Arabia

**Keywords:** Organ donation, Islamic ethics, Preference, Norm perception, Mandated choice, Presumed consent, Informed consent, Medical incentives, Financial incentive, Gender difference

## Abstract

**Background:**

Posthumous organ procurement is hindered by the consenting process. Several consenting systems have been proposed. There is limited information on public relative attitudes towards various consenting systems, especially in Middle Eastern/Islamic countries.

**Methods:**

We surveyed 698 Saudi Adults attending outpatient clinics at a tertiary care hospital. Preference and perception of norm regarding consenting options for posthumous organ donation were explored. Participants ranked (1, most agreeable) the following, randomly-presented, options from 1 to 11: no-organ-donation, presumed consent, informed consent by donor-only, informed consent by donor-or-surrogate, and mandatory choice; the last three options ± medical or financial incentive.

**Results:**

Mean(SD) age was 32(9) year, 27% were males, 50% were patients’ companions, 60% had ≥ college education, and 20% and 32%, respectively, knew an organ donor or recipient. Mandated choice was among the top three choices for preference of 54% of respondents, with an overall median[25%,75%] ranking score of 3[2,6], and was preferred over donor-or-surrogate informed consent (4[2,7], p < 0.001), donor-only informed consent (5[3,7], p < 0.001), and presumed consent (7[3,10], p < 0.001). The addition of a financial or medical incentive, respectively, reduced ranking of mandated choice to 7[4,9], p < 0.001, and 5[3,8], p < 0.001; for donor-or-surrogate informed consent to 7[5,9], p < 0.001, and 5[3,7], p = 0.004; and for donor-only informed consent to 8[6,10], p < 0.001, and 5[3,7], p = 0.56. Distribution of ranking score of perception of norm and preference were similar except for no-organ donation (11[7,11] *vs.* 11[6,11], respectively, p = 0.002). Compared to females, males more perceived donor-or-surrogate informed consent as the norm (3[1,6] *vs.* 5[3,7], p < 0.001), more preferred mandated choice with financial incentive option (6[3,8] *vs.* 8[4,9], p < 0.001), and less preferred mandated choice with medical incentive option (7[4,9] *vs.* 5[2,7], p < 0.001). There was no association between consenting options ranking scores and age, health status, education level, or knowing an organ donor or recipient.

**Conclusions:**

We conclude that: 1) most respondents were in favor of posthumous organ donation, 2) mandated choice system was the most preferred and presumed consent system was the least preferred, 3) there was no difference between preference and perception of norm in consenting systems ranking, and 4) financial (especially in females) and medical (especially in males) incentives reduced preference.

## Background

Organ transplantation has enabled many patients to have longer and better quality of life, which resulted in an increased demand for a limited supply of organs [1]. Posthumous donation is still the most important source of organs, and studies have shown that one of the major barriers to posthumous organ procurement is failure to obtain consent [2]. For instance, during the year 2008 in Saudi Arabia, 533 potential organ donors were identified, 282 families were approached, and only 118 families gave consent; and organ retrieval was carried out in 89%of consenting cases [3].

Short of mandated “donation” or conscription, several consenting systems have been proposed/used. Informed (or explicit or ‘opting in’) consent by the donor or his/her family is used in several countries worldwide including Saudi Arabia [3,4]. A presumed consent or ‘opting out’ system which enables hospitals to procure organs from potential donors unless the deceased has formally registered an objection [5], is used in most countries in Europe with a spectrum of enforcement and family involvement [4,5]. Unenforced presumed consent policies have the caveat of allowing family to refuse [6]. In a mandated choice system, competent adults are obliged to decide whether they wish to donate or not donate their organs after their deaths [7].

Organ donation has been generally based on altruistic donation, however, incentives have also been considered as a means to increase donation rates [2]. Financial incentives, any material gain or valuable consideration obtained by those directly consenting to the process [8], can be in the form of cash payments, contributions to burial expenses, tax breaks, health insurance for the immediate family, college scholarship for children, or donation to a charity of the donor choice [2,9]. Financial incentives are often disfavored because of potential connection to commercial trade. Medical incentives, rewarding donors by an in kind recognition, can be in the form of receiving points that enhance the likelihood of receiving an organ should it be needed [1].

Public opinion regarding the various consenting systems is crucial, is expected to be culture-specific [10], and requires further investigation [11]. One needs to determine the moral focus of the public since the introduction of a consenting system without public support could negatively affect donation rates. For example, preference for presumed consent may have basis in religious culture, as most Catholic as compared to Protestant countries presumes consent [12]. There are more than 1.3 billion Muslims worldwide [13]. Most Islamic scholars have accepted brain death as true death [3,13], and that organ donation is allowed if it is done with respect to the deceased and for the benefit of the patient [13,14]. However, the most suitable consenting system has not been clearly identified in Islamic cultures. Further, we are not aware of studies that directly compared public relative preference of the various consenting systems or that directly compared preference to perception of norm.

The aim of this study was to survey the Saudi public preference and perception of norm on several consenting systems for posthumous organ donation and determine if they are related to demographic data.

## Methods

This cross sectional survey was based on a convenience sample of a tertiary care hospital attendees and was conducted in accordance with the ethical principles contained in the Declaration of Helsinki and after approval of the Research Ethics Committee (REC) of the King Faisal Specialist Hospital and Research Center (KFSH&RC) in the period from November 2007 to November 2010. A request for waiver of written informed consent was approved by the REC and all respondents gave verbal informed consent.

Saudi adult individuals in the waiting areas of the outpatients’ clinics were approached by research coordinators. The number of individuals invited from each waiting area was prorated based on individual clinic load. The questionnaire was self-administered in Arabic language with research coordinator’s support as requested by respondents (the 14 illiterate respondents completed the questionnaire verbally). The following demographic data were collected, age, gender, reason of visit (clinic appointment, patient companion), perceived health status (healthy, ill), education level (illiterate, primary school, secondary school, college or higher), knowing an organ donor (yes, no), and knowing an organ recipient (yes, no).

The questionnaire was developed by the authors in Arabic language based on literature review. After initial development, the questionnaire was presented for comments to 5 physicians and revised accordingly (minor changes in language usage to have consistency throughout the questionnaire). Face validity was assessed by interviewing 10 respondents after completing the questionnaire. The final version was pilot-tested on 10 other respondents for acceptability, comprehensibility in the setting, clarity, and stability (2–3 days); and found suitable. The pilot results were not included in this report. An English translation (accuracy confirmed by back translation) of the questionnaire and the instructions given to respondents are available in the Additional file 1. The questionnaire consisted of two parts: the first part on personal preference and the second on perception of norm (what is perceived as best for the Saudi society, regardless of personal preference). The personal preference part presented participants with 11 consenting options: no-organ donation, presumed consent, informed consent by donor-only, informed consent by donor-or-surrogate, and mandatory choice; the last three options without or with medical incentive (if you register your willingness to donate your organ(s) after death, you and your family will be prioritized for receiving organs from other donors) or financial incentive (if you register your willingness to donate your organ(s) after death, or your family authorizes the donation of your organs after your death, a financial donation to your (or your family’s) charity of choice will be given by a third party). Similar statements with appropriate modifications were used for the second questionnaire part on perception of norm. The 11 options in the two parts were arranged in the same order for a given respondent. However, they were presented to respondents in a random order. Respondents were asked to rank the options from 1 (most preferred or best) to 11 (least preferred or worst). Participants were given the following introductory information: “This study is approved by the Research Ethics Committee of King Faisal Specialist Hospital & Research Center. It aims to explore the opinions and preferences of the Saudi public regarding the various consenting options for organ donation. The results of this study are expected to enlighten policy makers about the views of the Saudi public. Islamic scholars in Saudi Arabia have declared that organ donation is consistent with Islamic teaching. The Kingdom of Saudi Arabia has an active cadaveric transplant program. However, organs are in shortage and more than 3000 Saudi patients are waiting for organs. The shortage in organs is due in large part to the consenting process. Completing the questionnaire will take about 10–20 min. Your answers will not be linked to you and will not be used as your decision for organ donation. The aim of the study is to get the overall preference/opinion of Saudis rather than individual views. Below are several consenting options that are practiced in different parts of the world. A brief explanation is provided for each. If it is still not clear to you, please feel free to ask. We would like to request you to rank the options twice. First according to what you personally prefer and second, according to what you think would be best for the Saudi society in general (regardless of what you prefer for yourself). Thank you for choosing to take part in this study”.

The study was exploratory, and the sample size and sampling method were conveniently determined. The response rate was calculated as the number of usable questionnaires divided by the number of individuals approached. Data were verified by double entry and validity checks were undertaken. The mean (SD) and median [25% and 75%] score for each consenting option was determined. We used Kendall’s W test to compare median ranking scores among 3 consenting options (without or with financial or medical incentive) and Wilcoxon signed ranks test for pairwise comparisons. We used the Mann–Whitney test to examine if median ranking scores differed according to gender, health status, reason for visit, or knowledge of an organ donor or recipient; and the Kruskal-Wallis test to examine if they differed according to education level. Correlation between age and ranking scores of each option and between consenting options was studied using Spearman’s test. A 2-tailed p value of <0.01 was considered significant. Analyses were conducted by one of the author (MMH) with SPSS for Windows software (release 17.0.0, 2008. SPSS Inc., Chicago, ILL, USA). 2-tailed p values are reported.

## Results

1003 individuals were approached; 46 refused to participate, 60 did not understand the study, and 199 did not return a usable questionnaire. The main reasons for not completing the questionnaire were: people were expecting to be called for their appointment, were occupied with children, did not feel well, or did not want to be bothered. Thus responses from 698 (70%) individuals were available for analysis. The demographics of 698 respondents are shown in Table 1. Demographic data of subjects who did not understand the study or did not return a useable questionnaire were statistically different from those of the respondents only in having older age (mean (SD), 35(10) year), lower percentage of males (15%), and lower education level (33% with college or higher education). Each of the 11 options was ranked by 629 (90%) to 661 (95%) of the respondents for personal preference and by 593 (85%) to 612 (88%) for perception of norm.

**Table 1:** Characteristics of study participants (no. = 698)

**Age-mean (SD), yr**	**32 (9)**
**Gender-no. (%)**	
Male	187 (27)
Female	506 (73)
**Know organ donor?-no. (%)**	
No	551 (80)
Yes	141 (20)
**Know organ Recipient?-no. (%)**	
No	472 (68)
Yes	219 (32)
**Education Level-no. (%)**	
Illiterate	14 (2)
Primary school	35 (5)
Secondary school	228 (33)
College or higher	416 (60)
**Purpose of visit-no. (%)**	
Clinic appointment	339 (50)
Patient companion	337 (50)
**Health status-no. (%)**	
Healthy	539 (78)
Ill	149 (22)

Numbers don’t add up to 698 because some entries were not completed by some respondents.

### Objection to posthumous organ donation

655 respondents to the preference questionnaire ranked the no-organ donation option. 64% assigned to this option the last 3 ranks (9–11), 18% the five middle ranks (4–8), and 17% the first 3 ranks (1–3), with a median [25%, 75%] ranking score of 11 [6,11], indicating that the majority of respondents favored organ donation (Table 2).

**Table 2:** Personal preference of eleven consenting options

	**No-organ donation (655)**	**Mandated choice (661)**	**Mandated choice + financial incentive (637)**	**Mandated choice + medical incentive (649)**	**Donor-only informed consent (655)**	**Donor- only informed consent + financial incentive (629)**	**Donor- only informed consent + medical incentive (645)**	**Donor-or- surrogate informed consent (648)**	**Donor-or- surrogate informed consent + financial incentive (637)**	**Donor-or- surrogate informed consent + medical incentive (649)**	**Presumed consent (649)**
**1**^**st**^**Rank**	13	23	4	14	12	3	9	13	2	10	8
**2nd Rank**	2	18	7	11	11	4	13	15	6	8	9
**3rd Rank**	2	13	10	9	13	5	9	13	5	14	8
**4th Rank**	2	9	7	11	9	6	12	13	7	13	12
**5th Rank**	4	7	6	8	13	6	15	7	8	17	7
**6th Rank**	2	9	8	11	10	7	12	13	9	12	4
**7th Rank**	5	5	9	9	8	11	11	10	14	8	8
**8**^**th**^**Rank**	5	5	14	8	6	16	9	5	16	9	4
**9th Rank**	4	6	16	9	9	15	6	6	17	5	4
**10**^**th**^**Rank**	10	3	13	7	7	17	4	4	13	2	17
**11th Rank**	50	2	5	2	1	11	1	1	3	2	19
**Median [25%, 75%]**	11 [6,11]	3 [2,6]	7 [4,9]	5 [3,8]	5 [3,7]	8 [6,10]	5 [3,7]	4 [2,7]	7 [5,9]	5 [3,7]	7 [3,10]
**Mean [SD]**	8.3 [3.7]	4.0 [2.8]	6.6 [2.9]	5.2 [3.0]	5.0 [2.8]	7.5 [2.7]	5.1 [2.6]	4.6 [2.7]	7.0 [2.5]	5.0 [2.5]	6.6 [3.5]

Numbers between () represent the number of responses. Data indicate the percentage of respondents who assigned the indicated rank to the corresponding consenting option. Wilcoxon signed ranks test: mandated choice *vs.* donor-only informed consent, p <0.001; mandated choice *vs.* donor-or-surrogate informed consent, p <0.001; mandated choice *vs.* presumed informed consent, p <0.001. Kendall’s W coefficient of concordance (comparing mandated choice, donor-only informed consent, or donor-or-surrogate informed consent each with the same option plus medical or financial incentives) was, 0.115, 0.189, and 0.183, respectively (p <0.001).

The distribution of perception of norm ranking scores followed the same pattern (Table 3) and there was significant correlation between personal preference and perception of norm (rho 0.68, P <0.001). Nevertheless, there was significant difference between the two distributions (11[6,11] *vs.* 11[7,11], respectively, p = 0.002), indicating that even a higher percentage of respondents perceived organ donation as the norm.

**Table 3:** Perceived norm of eleven consenting options

	**No-organ donation (608)**	**Mandated choice (612)**	**Mandated choice + financial incentive (596)**	**Mandated choice + medical incentive (604)**	**Donor-only informed consent (605)**	**Donor- only informed consent + financial incentive (596)**	**Donor- only informed consent + medical incentive (601)**	**Donor-or- surrogate informed consent (605)**	**Donor-or- surrogate informed consent + financial incentive (593)**	**Donor-or- surrogate informed consent + medical incentive (595)**	**Presumed consent (603)**
**1st Rank**	8	19	6	11	12	4	8	13	3	10	13
**2nd Rank**	2	18	8	13	11	5	12	14	4	8	8
**3rd Rank**	2	12	12	11	11	5	10	11	6	12	8
**4th Rank**	3	11	7	12	11	8	11	12	8	9	11
**5th Rank**	3	7	7	12	9	8	14	8	7	16	7
**6th Rank**	2	7	8	10	12	8	13	12	9	14	4
**7th Rank**	6	7	9	9	8	11	9	9	11	12	8
**8th Rank**	5	5	16	7	7	13	10	6	16	8	6
**9th Rank**	4	8	13	9	7	14	8	7	17	7	4
**10th Rank**	10	4	12	5	8	15	4	4	15	4	16
**11th Rank**	54	2	4	1	3	10	1	3	3	1	16
**Median [25%, 75%]**	11 [7,11]	4 [2,7]	7 [3,9]	5 [3,7]	5 [3,8]	8 [5,9.4]	5 [3,7]	4 [2,7]	8 [5,9]	5 [3,7]	6 [3,10]
**Mean [SD]**	8.8 [3.3]	4.3 [2.9]	6.2 [3.0]	5.0 [2.8]	5.3 [3.0]	7.1 [2.9]	5.2 [2.6]	4.9 [2.8]	7.0 [2.6]	5.1 [2.5]	6.3 [3.6]

Numbers between () represent the number of responses. Data indicate the percentage of respondents who assigned the indicated rank to the corresponding consenting option. Wilcoxon signed ranks test: mandated choice *vs.* donor-only informed consent, p <0.001; mandated choice *vs.* donor-or-surrogate informed consent, p <0.001; mandated choice *vs.* presumed informed consent, p <0.001. Kendall’s W coefficient of concordance (comparing mandated choice, donor-only informed consent, or donor-or-surrogate informed consent each with the same option plus medical or financial incentives) was 0.077, 0.130, and 0.150, respectively (p <0.001).

### Preferred posthumous consenting option

**Figure 1:**
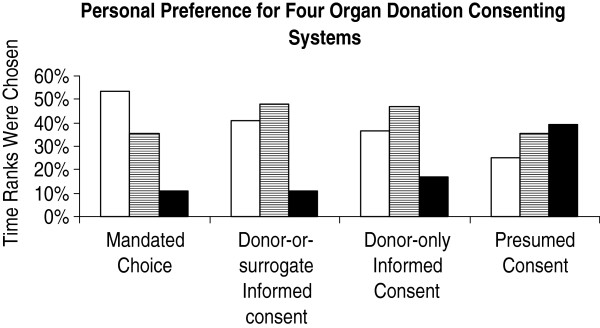
**Personal preference for four organ donation consenting systems.** Open bars indicate the percentage of time the highest ranking scores (1–3) were chosen. Bars with horizontal lines indicate intermediate ranking scores (4–8) and black bars lowest ranking scores (9–11).

As shown in Table 2 and Figure 1, mandated choice option was assigned the top three ranks by 54% of respondents, with an overall median score of (3 [2,6]), and was preferred over the options of donor-or-surrogate informed consent (4 [2,7], p <0.001), donor-only informed consent (5 [3,7], p <0.001), and presumed consent (7 [3,10], p <0.001). Further, donor-or-surrogate informed consent, donor-only informed consent, and presumed consent options were assigned the top three ranks by 41%, 36%, and 25% of respondents, respectively. Donor-or-surrogate informed consent and donor-only informed consent were assigned the five intermediate ranks by a larger percentage of respondents (48% and 46%, respectively) as compared to mandated choice and presumed consent options (35%, respectively), indicating a clearer choice for the last two at the public level.

The distribution of ranking scores of perception of norm followed the same preference pattern (Table 3), and there was significant correlation between personal preference and perception of norm for mandated choice (rho 0.51, p <0.001), donor-or-surrogate informed consent (rho 0.60, p <0.001), donor-only informed consent (rho 0.56, p <0.001), and presumed consent (rho 0.61, p <0.001). Further, there was no significant difference between personal preference and perception of norm in median ranking score for mandated choice (3 [2,6] *vs.* 4 [2,7], respectively, p = 0.09), donor-or-surrogate informed consent (4 [2,7] *vs.* 4 [2,7], respectively, p = 0.04), donor-only informed consent (5 [3,7] *vs.* 5 [3,8], respectively p = 0.31), presumed consent options (7 [3,10] *vs.* 6 [3,10], respectively, p = 0.02), mandated choice with medical incentive (5 [3,8] *vs.* 5 [3,7], respectively, p = 0.04), donor-or-surrogate informed consent with financial (7 [5,9] *vs.* 8 [5,9], respectively, p = 0.86), or medical incentive (5 [3,7] *vs.* 5 [3,7], respectively, p = 0.26), or donor-only with medical incentive (5 [3,7] *vs.* 5 [3,7], respectively, p = 0.23). However, there was significant difference between personal preference and perception of norm in ranking score for mandated choice with financial incentive (7 [4,9] *vs.* 7 [3,9], respectively, p = 0.001) and donor-only informed consent with financial incentive (8 [6,10] *vs.* 8 [5, 9.8], respectively, p <0.001), indicating higher acceptance of the addition of financial incentive as the norm (*vs.* personal preference).

### The effect of adding incentives

The addition of a financial incentive reduced the preference for mandated choice option from 3 [2,6] to 7 [4,9] (p <0.001), for donor-or-surrogate informed consent from 4 [2,7] to 7 [5,9] (p <0.001), and for donor-only informed consent from 5 [3,7] to 8 [6,10] (p <0.001).

**Figure 2:**
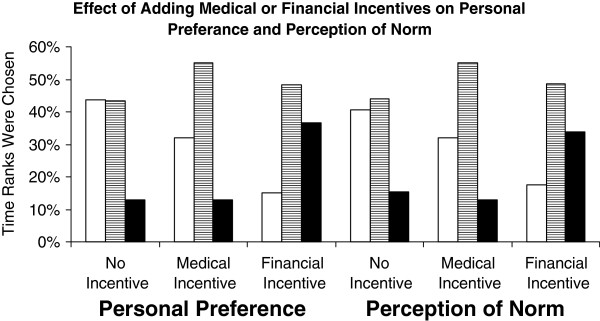
**Effect of adding medical or financial incentives to consenting options on personal preference and perception of norm.** Open bars indicate the percentage of time the highest ranking scores (1–3) were chosen. Bars with horizontal lines indicate intermediate ranking scores (4–8) and black bars lowest ranking scores (9–11).

The addition of a medical incentive had a similar but smaller affect. It reduced the preference for mandated choice option to 5 [3, 8] (p <0.001), for donor-or-surrogate informed consent to 5 [3,7] (p = 0.004), but not for donor-only informed consent (to 5 [3, 7], p = 0.56). Figure 2 compares financial and medical incentives to no incentive after combining data for the three consenting options. It shows that the two incentives differ not only by the degree of their negative effect but also by the way they affect the distribution of ranking scores. Medical incentive reduced overall preference score by increasing intermediate ranking, whereas financial incentive reduced preference mainly by increasing less favorable ranking. The effect of the two incentives on perception of norm was in general similar to their effect on personal preference (Figure 2). However, as indicated above, adding financial incentive had more detrimental effect on personal preference than perception of norm when added to mandated choice or donor-only informed consent.

### Association between responses and respondents’ demographics

There was no significant correlation between ranking scores for each of the 11 consenting options and age (for personal preference: p value ranged from 0.01 (rho 0.17) for no-organ donation to 0.94 for donor-only informed consent with financial incentive; for perception of norm: p value ranged from 0.11 for no-organ donation to 0.98 for donor-or-surrogate informed consent with financial incentive).

There was no difference between subgroups based on perceived health status (for personal preference: p value ranged from 0.05 for mandated choice with medical incentive to 0.96 for mandated choice with financial incentive; for perception of norm: p value ranged from 0.20 for mandated choice to 0.91 for mandated choice with financial incentive), or reported reason for hospital visit (for personal preference: p value ranged from 0.11 for mandated choice with medical incentive to 0.96 for no-organ donation; for perception of norm: p value ranged from 0.16 for donor-or-surrogate informed consent with financial incentive to 0.94 for presumed consent), or education level (for personal preference: p value ranged from 0.17 for presumed consent to 0.88 for donor-only informed consent with medical incentive; for perception of norm: p value ranged from 0.11 for donor-only informed consent to 0.88 for no-organ donation).

There was no significant difference between subgroups based on knowing an organ donor (for personal preference: p value ranged from 0.14 for donor-or-surrogate informed consent to 1.0 for donor-only informed consent with financial incentive; for perception of norm: p value ranged from 0.08 for mandated choice to 0.83 for donor-only informed consent) except of border line significance for presumed consent option (7 [4,10] for respondents who did not know an organ donor *vs.* 5 [3,10] for those who did, p = 0.03) and no-organ donation option (10 [6, 11] *vs.*11 [8, 11], respectively, p = 0.03). Similarly, there was no significant difference between subgroups based on knowing an organ recipient (for personal preference: p value ranged from 0.51 for donor-only informed consent with medical incentive to 1.0 for mandated choice with medical incentive; for perception of norm: p value ranged from 0.21 for no-organ donation to 0.94 for donor-only informed consent).

Gender had several significant associations (Table 4). Compared to females, males more preferred mandated choice with financial incentive option (6 [3,8] *vs.* 8 [4,9], p <0.001), less preferred mandated choice with medical incentive option (7 [4,9] *vs.* 5 [2,7], p <0.001), and more perceived donor-or-surrogate informed consent as the norm (3 [1,6] *vs.* 5 [3,7], p <0.001). Mandated choice option continued to be significantly the most preferred and best perceived as norm and financial and medical incentives continued to significantly have a negative effect when analysis was restricted to females. Similar results were obtained when analysis was restricted to males, except that the significant difference between mandated choice and donor-or-surrogate informed consent options was lost both for personal preference (p = 0.17) and perception of norm (p = 0.27).

**Table 4:** Personal preference and perception of norm of eleven consenting options according to gender

	**No-organ donation**	**Mandated choice**	**Mandated choice + financial incentive**	**Mandated choice + medical incentive**	**Donor-only informed consent**	**Donor-only informed consent + financial incentive**	**Donor-only informed consent + medical incentive**	**Donor-or- surrogate informed consent**	**Donor-or- surrogate informed consent + financial incentive**	**Donor-or- surrogate informed consent + medical incentive**	**Presumed consent**
**Males**											
**Personal Preference**^**a**^	11 [7,11]	3 [2,6]	6 [3,8]	7 [4,9]	5 [3,7]	8 [5,10]	5 [3,7]	4 [2,6]	7 [5,9]	5 [3,7]	6 [4,10]
**No. percentile)**	176	175	171	171	176	167	171	174	170	170	174
**Perception of Norm**^**b**^	11 [7.8,11]	3.5 [2,7]	7 [3,8]	5 [3,8]	5 [3,8]	7.5 [4,9]	6 [3, 7]	3 [1,6]	7 [5, 9]	5 [4,7]	7 [4,10]
**No.**	162	160	157	159	160	154	157	162	156	154	159
**Females**											
**Personal Preference**^**c**^	10 [6,11]	3 [2,6]	8 [4,9]	5 [2,7]	5 [3,7]	8 [6,10]	5 [3,7]	4 [2,7]	8 [5,9]	5 [3,7]	7 [3,10]
**No.**	474	481	461	473	474	457	469	469	462	474	470
**Perception of Norm**^**d**^	11 [7,11]	4 [2,7]	7 [4,9]	5 [2,7]	5 [3,8]	8 [5,10]	5 [3 7]	5 [3,7]	8 [5,9]	5 [3,7]	6 [3,10]
**No.**	443	449	435	442	442	438	441	440	433	438	441

Data indicate median [25, 75 percentile]. a, Wilcoxon signed ranks test: mandated choice *vs.* donor-only informed consent, p <0.001; *vs.* donor-or-surrogate informed consent, p = 0.17; *vs.* presumed informed consent, p <0.001; *vs.* mandated + financial incentive, p <0.001; and *vs.* mandated + medical incentive, p <0.001. b, Wilcoxon signed ranks test: mandated choice *vs.* donor-only informed consent, p = 0.002; *vs.* donor-or-surrogate informed consent, p = 0.27; *vs.* presumed informed consent, p <0.001; *vs.* mandated + financial incentive, p <0.001; and *vs.* mandated + medical incentive, p = 0.004. c, Wilcoxon signed ranks test: mandated choice *vs.* donor-only informed consent, p <0.001; *vs.* donor-or-surrogate informed consent, p <0.001; *vs.* presumed informed consent, p <0.001; *vs.* mandated + financial incentive, p <0.001; and *vs.* mandated + medical incentive, p <0.001. d, Wilcoxon signed ranks test: mandated choice *vs.* donor-only informed consent, p <0.001; *vs.* donor-or-surrogate informed consent, p = 0.17; *vs.* presumed informed consent, p <0.001; *vs.* mandated + financial incentive, p <0.001; and *vs.* mandated + medical incentive, p = 0.003.

## Discussion

The aims of this study were to survey Saudi public preference and perception of norm on several consenting options for posthumous organ donation and determine if they are associated with certain demographic data. We studied a convenient sample of 698 adults in the outpatient setting at a tertiary healthcare center in Riyadh, Saudi Arabia. The study sample had a mean (SD) age of 32 (9) year, 27% were males, and 60% had college or higher education. The strengths of the study include a relatively large sample size, a high response rate, simultaneous examination of preference and perception of norm, directly comparing various consenting options, and uniquely addressing Islamic/Arab culture. We found that: 1) most respondents were in favor of posthumous organ donation, 2) mandated choice system was the most preferred and presumed consent system was the least preferred, 3) there was no difference between preference and perception of norm in consenting systems ranking, and 4) financial (especially in females) and medical (especially in males) incentives reduced preference for mandated choice and informed consent options.

In trying to adopt a consenting system for posthumous organ donation, the following considerations are relevant.

1) A common notion is that living people may have interests in the future when they have ceased to exist; although experiential interests are fulfilled only during life, critical interests, such as interest in a good reputation, confidentiality, and body organs after death, can be fulfilled after death [15,16]. Such critical interests may matter because of their importance for people when they were alive, to avoid psychological injury to family, and to promote socially desirable behavior.

2) Is the body a property? One of the fundamental rights is self ownership; the right of non-interference in one’s body without consent. In certain situations, this right is extended to accept payment for body usage (for example, when remuneration is offered for participation in research), and it has been argued that body organs are akin to goods to which one can claim rights [17,18]. However, it is clear that people don’t own their bodies in the way they own their homes. In contrast to natural rights theory, social constructivist theory hold that property is a socially constructed bundle of separable social relations rather than an indivisible unit, and that ownership is a legal relation between the owner and non-owners (rather than between the owner and the owned subject). Thus the issue of property rights to organs should not be reduced to a simple binary issue of owning and not owning [19].

3) Who “owns” the body of the deceased? The family has a limited property rights in the body of the deceased according to US court [20]. Since the sense of body ownership is related to the interest of what happen to the body rather than to financial transaction, it is difficult to think of owning one’s body after death. On the other hand, the communitarian approach (concept of reciprocity) suggests that organ donation is an act of paying back of an obligation to the community [12]. Do individuals’ critical interests in the disposition of their organs after death trump the experiential interest of family members or the experiential interest of organ recipients?

4) The magnitude of psychological harm from an erroneous donation under presumed consent may be more than that from an erroneous non-donation under informed consent; the disutility of losing may be more than the disutility of not getting, and expressing opposition may reflect deeper commitment than expressing agreement.

5) People may be more likely to donate when they feel they retain control and people may not object to the act of donation but to the consenting system itself. An act that is freely chosen, regardless of whether it is objectively wrong, may have a greater moral value than an act that may be objectively good but has not been freely chosen [7,20,21].

### Diversity in preference and perception of norm

The observed diversity in preferences suggests that a one-size-fits-all policy on posthumous organ donation may results in some degree of public dissatisfaction. The diversity in perceptions of norm may be due to an absence of a norm, that the norm is not well known to the public, or that there are several rather than a single norm. The later is more likely. There is of course no statement in Quran or Prophet Muhammad’s Sayings that directly address organ donation, consequently positions on organ donation are based on interpretation. The Saudi Senior Ulama Commission decree issued in 1982 permitted organ donation and transplantation from living and deceased donors [3]. However, although most current Islamic scholars are in favor of posthumous organ donation, some disagree. Further, there is disagreement among who allow organ donation and whether it is obligatory, encouraged, or just permitted. Based on interviews with the main faith and belief organizations (including Islamic) within the UK, it was found that none was against organ donation in principle, that the majority opinion in each faith group permit organ donation, and that there is a broad spectrum of opinions within each group [22]. A 1996 study conducted in Saudi Arabia found that 56% believed that Islam permits transplant and 31% did not know [23] and a 2005 study found that 29% believed that Islam permits transplant and 24% did not know [24]. Similarly, a study on students from the faculty of theology in Turkey found that 16.5% thought that organ donation is not in accord with Islamic beliefs [25]. A more recent study in Pakistan found that that the belief that organ donation is allowed in religion was a significant independent predictor for willingness to donate [26].

### Preference for organ donation

Understanding cultural expectations can provide insight into people preferences and perceptions. Just as secular Western societies continue to be influenced by Judo-Christian norms concerning social ethics [27], Arabic and Islamic societies are still influenced by Islamic social ethics which shares many foundational values with Judaism and Christianity [28]. Saving life and helping others are praised in several verses of Quran, for example, “and if any one saved a life, it would be as if he saved the life of the whole people.” (Chapter 5, verse 32) [29].

We found that only 13% and 8% of respondents selected the option of no-organ donation as the first choice for personal preference and perceived norm, respectively. This is consistent with previous studies in Saudi Arabia [23,24,30,31] and other Islamic countries [25,26,32-35]. In Saudi Arabia, a 1991 study found that 53% of responders either signed a kidney donor card or expressed willingness to do so [30], a 1996 study found that 67% were willing to donate [23], a 2005 study found that 42% agreed to donate [24], and a 2009 study found that 71% were willing to donate [31]. A 2005 study in Qatar, found that 37.8% of Qataris and 32.8% of non-Qataris were willing to donate [32], a 2009 study in Pakistan found that 62% expressed a motivation to donate [26], a 2006 study in Nigeria showed that 30% expressed a willingness to donate [33], a 2009 study in Malaysia showed that 41% reported that they have registered to be organ donors or indicated willingness to donate [34], and in Turkey, a 2002 study found that 57% were willing to donate [35] and a 2009 study of students from the faculty of theology found that 24% were willing to donate their organs and 57% were undecided [25].

It is not known if the degree of the expressed preference for donation would change during illness or impending death. Interestingly, we found no difference between subgroups based on perceived health status or reported reason for hospital visit (having a clinic appointment *vs.* being a companion) in personal preference or perception of norm.

### Mandated choice system was the most preferred

We found that the most favorable system (both from the point of personal preference and the point of perception of norm) for consenting for posthumous organ donation was the mandated choice system. In this line, a survey of young adults in the USA indicated that 90% supported mandated choice (*vs.* 60% for presumed consent) [36]. Further, the UK Royal College of Physicians has called for a system of mandated choice [37], which was also the preferred option of the American Medical Association and the United Network for Organ Sharing (UNOS) but not the British Medical Association [7].

Mandated choice system falls between informed consent system on one hand and presumed consent and mandated donation systems on the other. Advocates of mandated donation system, a system based on the notion of normative consent (it is immoral for an individual to refuse consent) [38], and the belief that the body should be considered as “on loan” to the individual from the biomass [39], argue that people should not be permitted a choice in this matter. It is counter argued that choosing not to save someone’s life is not the same as murder, and that although utilitarianism makes no distinction between causing an event and allowing it to happen when it was physically within our power to prevent [40], people (and deontologist) differentiate between intended harm and foreseen harm [21]. Further, mandated donation system would remove the moral content of organ donation since beliefs and desires matter for moral judgments (e.g., we forgive accidental harms and condemn failed attempts to harm) [21]. Furthermore, there are surviving (or persisting or critical interests) of the dead that should be respected [15,16,40]. Advocates of informed consent system argue that compelling people to choose may undermine autonomy because it constitutes a coerced burden. However, mandated choice does promote autonomy, from the point of view that it ensures that one’s preference is respected, and the coerced burden is not dissimilar to the duty of easy rescue (low burden that makes a great difference). In this line, not helping others when the cost to the helper is trivial is condemned in Quran, “So woe to the worshippers, Who are neglectful of their prayers, Those who (want but) to be seen (of men), But refuse (to supply) (even) neighborly needs (Al-Ma'un, small kindnesses e.g. salt, sugar, water, etc.).” (Chapter 107, verses 4–7) [29]. It has been argued that if respect for individual autonomy is the greatest concern, then mandated choice is preferred. If ensuring an adequate supply is considered to be most important, and mandated choice is unable to achieve the goal, then conscription is the best approach [41].

The implementation of a mandated choice system may not be easy as it requires a centralized data bank and may reduce organ availability; it was tested in Texas during the 1990’s, when forced to choose, almost 80% of the people chose not to donate [42]. A mandated choice where the accompanying public education is pro donation has been recommended [7].

### Presumed consent system was disfavored

We found that the presumed consent system is the most disfavored among the options studied (we did not explore family involvement or differentiate between stringent and lenient systems). A systemic review of 8 attitude surveys of the UK public to presumed consent reported 28-57% support before 2000, which increased to 64% in 2007 [42]. Surveys from other countries, showed that only in Belgium was there an overall approval of presumed consent [43]. The majority of faith and belief leaders in the UK was supportive of the opt-in system, and favored retaining it over the introduction of an opt-out system [44]. Presumed consent was not supported by the Institute of Medicine and was rejected by the American Medical Association’s Council on Ethical and Judicial Affairs; however, the British Medical Association produced a report supporting it [7].

The presumed consent system has been the subject of major public and ethical debates because it put the utilitarian and rights and justice approaches to ethics in conflict. It is associated with higher donation rate [4,45], and the association may be causative [4], however, the extent of which has been debated [45]. Presumed consent system may represent a violation of the right of autonomy, where the individual’s body would become public puberty unless claimed otherwise [46], and it has been argued that is not really an informed consent (as there may be newer procedures that were not envisioned by the patient at the time the intent was expressed) and that silence can be a sign of ambivalence and confusion rather than willingness [47]. Further, it may be considered by some as inaccurate and misleading; unlike the presumptions in law and science, presumption of consent cannot afford any possibility of reversing the decision or retracting any action based on the decision [11]. Furthermore, vulnerable populations such as minority cultural groups and immigrants may be less likely to support donation, less likely to realize that a presumed consent system exists, and more likely to find it challenging to opt out of donating [42].

The expressed disfavoring of presumed consent that we found in our study could be due to distrust in the medical system (people may feel that less effort will be made to keep them alive, that their body will be mutilated) and to the feeling of losing control. Additionally, based on the virtue approach to ethics, one need to will the good act [21,48]. Islamic teachings emphasize the importance of the intention and will. Prophet Muhammad said, "The reward of deeds depends upon the intentions and every person will get the reward according to what he has intended” (Sahih Albukhari Volume 1, Book 1, Number 1) [49]. The fact that the respondents disfavored incentives suggests that they favor donation for altruistic reason. Since altruism requires wanting and willing the act, one would expect that presumed consent will be disfavored.

### Negative effect of added incentives

We found that adding a medical or financial incentive to a mandated choice, donor-only, or donor-or-surrogate system had a negative effect both from preference, and to a lower extent, norm perception points of view. Adding financial incentive had more negative effect than adding medical incentive, which was mainly due to increased less favorable ranking rather than intermediate ranking.

Organ donation has long relied on altruism. However, financial incentives have been advocated [50]. The majority of members of the American Society of Transplant Surgeons supported funeral reimbursement or charitable organization donation [51] and the Council on Ethical and Judicial Affairs (1995) of the American Medical Association has recommended that an empirical trial of financial rewards for organ donors should be conducted to determine its impact on overall donation rate [52].

Arguments in favor of adding incentives include an increase in organ supply based on basic economics [1], intrinsic fairness with regard to opting in, and that failure to allow incentives interferes with individual anatomy. It is of note that the current system is based on gain for all concerned, except the donor who makes the sacrifice. Arguments against adding incentives include that it still represents compensation akin to purchase and thus can negatively affect altruistic culture and lead to exploitation of lower income groups, that it results in decreased respect for sanctity of human body [53], that its implementation is difficulty (the problem of cheap commitment), and that for in kind medical incentives, the fact that apart from an organ one needs health insurance to get a transplant, and that it favors larger families with more first-degree relatives. Further, willingness to donate might not necessarily increase donation rate if relatives can still decline organ donation [54]; individuals are more likely to donate their organs than to donate their deceased relatives’ organs [6], and inducements to register as an organ donor may distort the signal that registration makes about preferences (induce family members to impute a weaker preference) [55].

We are not aware of published public surveys on medical incentives. Consistent with our results, a 2005 study in Saudi Arabia found that only 0.6% of the respondents agreed to donate their organs after death for financial reasons [24] and a study in Scotland found that only 21% agreed that a financial incentive should be used [56]. However, lack of incentives was stated as a reason for not willing to donate by 14% of rural and 47 %urban Saudis [57], and 59% of respondents in Pennsylvania favored the general idea of incentives with 53% saying that direct payment would be acceptable [1,58]. The reason for disfavoring incentives in our study sample is not clear. Consistent with our observation that financial incentive was more dis-favored than medical incentive (and more sharply so), it is possible that people feels that donation is an act of charity that should be done purely for the sake of God and thus should not be compensated and that organs are not a property of the person and are too sacred to be exchanged for material benefits [59].

### No difference between preference and perception of norm

Although the public generally express favorable views toward organ donation [16,60,61], few actually take the necessary steps [6,16]. The gap between favorable opinion and actual behavior could be due to the difference between preference and perception of norm; individuals may express favor towards organ donation as an abstract concept for the society [6], whereas a statement of a preference is more a statement about the person who has the preference than the issue. Alternatively, the gap could be due to biased surveys or to obstacles (relative to the strength of preference) in converting a preference to an action (because of inertia and disutility of thinking about death) [6]. Our failure to find significant differences between preference and perception of norm suggests that the gap may be due to obstacles. However, such failure could be due to respondents’ inability to differentiate between the two. This is not likely because they were relatively highly educated (61% had college or higher education) and the two questions were presented at the same time. Alternatively, it may reflect a rather norm-desiring culture that seeks harmony between motives (preference) and reasons (perception of norm) or a social desirability bias (a low inclination to express a preference that is different from the perceived norm). Previous studies in the same population showed no significant difference between preference and perception of norm in regard to disclosure of medical errors [62] but not to consenting for research on left over tissue samples [63].

### Association with demographics

We found no significant correlation between ranking scores for each of the consenting options and age. However, older age was positively associated with ranking score for the no-organ donation option. This is consistent with previous studies showing that older people are less likely to donate and that younger age correlates positively with willingness to donate [6]. However, a study in Barbados, a middle income country in eastern Caribbean [64] found no association between age and attitudinal barriers. We found no difference between subgroups based on education level. This is in contrast to previous studies. Rural respondents in Saudi Arabia were less likely to report willingness to donate organs or to sign a donation card [57], and those who had finished their studies by the age of 15 were approximately half as willing to become a cadaveric donor as those who had completed additional schooling [6].

There is some evidence that individuals who find themselves increasingly likely to need an organ more intensively perceive the benefits of organ donation [6]*.* We found no difference between subgroups based on perceived health status, perceived reason for hospital visit, or reporting knowing an organ recipient. However, interestingly, respondents who reported knowing an organ donor more favored the presumed consent option and more disfavored no-organ donation option (borderline significance). It is of note that although 55%of the current study population had clinic appointment, 73%were self-perceived as healthy, which has been noted in a previous study and may reflect adaption to the state of illness [6].

Finally, we found that the addition of financial incentive has a more negative effect on females’ preference and perception of norm than on males. The addition of medical incentive has the opposite affect. This may reflect a difference in gender view of the two types of incentives based on current social role, financial incentives may be seen more amoral and medical incentives less amoral by females because females carry less financial responsibility and are less affluent than males. Females were also less likely to perceive donor-or-surrogate informed consent (but not donor-only informed consent) as the norm, maybe unconsciously stressing self-based (rather than family-based) decision making. Some [6] but not all [70] studies have shown that females may be more likely to donate.

### Study limitations

The study was based on convenience sampling and was performed in a single tertiary health care institution in a major metropolitan city and thus the results may not be generalizable to the general public. Further, the study sample overrepresented females and people with higher education. However, it is of note that the institution is a governmental referral center for the entire country, restricting analysis to males or females did not change the main conclusions of the study, and subgroup analysis based on education level did not reveal significant differences (though there was relatively small numbers in the lower education groups). Since public opinion regarding the various systems for posthumous organ donation would be expected to continue to evolve, the results may not be extrapolateable in time. The study also addressed preferences and perceptions rather than actual choices and did not include the option of family veto.

## Conclusions

In the setting of outpatient clinics at a tertiary care hospital in Saudi Arabia, we found that: 1) Most respondents favored posthumous organ donation. 2) There is a considerable diversity regarding the most favored consenting system, which may indicate that a one-fits-all policy may result in public’ dissatisfaction and that there is a need for more public education/debates. 3) Distribution of ranking scores of preference and perception of norm were similar, suggesting a rather norm-desiring culture. 4) A mandatory choice system was most favored and a presumed consent system was least favored, suggesting a combination of a response to the duty of easy rescue and the importance to will/intend the act of donation. 5) The addition of financial and medical incentives had negative effect, suggesting prevalence of altruistic motive and belief in sacredness of the body. 6) There was no association between favoring a consenting system and age, perceived health status, education level, or knowing an organ donor or recipient.

## Competing interests

The authors declare that they have no competing interests.

## Authors’ contributions

MMH designed the study, performed statistical analysis, performed in literature review, and wrote the manuscript. HMA, AE, HA, and AA participated in data collection. SH participated in statistical analysis and literature review and co-wrote the manuscript. KAC participated in study design and literature review. EAG participated in data collection and critically reviewed the initial draft. All authors read and approved the final manuscript.

## Pre-publication history

The pre-publication history for this paper can be accessed here:

http://www.biomedcentral.com/1472-6939/13/32/prepub

## Supplementary Material

Additional file 1 Consenting Options for Organ Donation: A Survey of the Opinions and Preferences of Saudis. Click here for file
