# Supplementary material for: Consenting options for posthumous organ donation: presumed consent and incentives are not favored
Source: BMC Med Ethics. 2012 Nov 22;13:32. doi: 10.1186/1472-6939-13-32 (PMC3519501; doi:10.1186/1472-6939-13-32)
Supplement: Additional file 1 — Consenting Options for Organ Donation: A Survey of the Opinions and Preferences of Saudis. [file 1472-6939-13-32-S1.doc]

**Consenting Options for Organ Donation:**

### A Survey of the Opinions and Preferences of Saudis

This study is approved by the Research Ethics Committee of King Faisal Specialist Hospital & Research Center. It aims to explore the opinions and preferences of the Saudi public regarding the various consenting options for organ donation. The results of this study are expected to enlighten policy makers about the views of the Saudi public.

Islamic scholars in Saudi Arabia have declared that organ donation is consistent with Islamic teaching. The Kingdom of Saudi Arabia has an active cadaveric transplant program. However, organs are in shortage and more than 3000 Saudi patients are waiting for organs. The shortage in organs is due in large part to the consenting process.

Completing the questionnaire will take about 10-20 minutes. Your answers will not be linked to you and will not be used as your decision for your organ donation. The aim of the study is to get the overall preference/opinion of Saudis rather than individual views.

Below are several consenting options that are practiced in different parts of the world. A brief explanation is provided for each. If it is still not clear to you, please feel free to ask us. We would like to request you to rank the options twice. First according to what you personally prefer and second, according to what you think would be best for the Saudi society in general (regardless of what you prefer for yourself).

Thank you for choosing to take part in this study.

**What you prefer for your self**

Please rank the following consenting options according to your own preference, i.e., what you prefer for yourself. There are five main options and two kinds of incentives that can be combined with three of them. The options add up to a total of 11. Use each number only once. Please read all the 11 options before you answer.

Go over the 11 options. Select the most preferred one. Give it number one. Then go over the 10 options left. Select the most preferred one of the 10 and give it number 2. Then go over the 9 options left, select the most preferred one and give it number 3. Continue until you rank all the options; 1 for the most preferred and 11 for the least preferred.

*Incentive A: If you register your willingness to donate your organ(s) after death, you and your family will be prioritized for receiving organs from other donors.*

*Incentive B: If you register your willingness to donate your organ(s) after death, or your family authorizes the donation of your organ(s) after your death, a financial donation to your (or your family’s) charity of choice will be given by a third party.*

_________No-organ Donation: I don’t want to donate any of my organs after my death.

_________Donor-only Informed Consent: You don’t have to decide whether you wish to donate (or not to donate) your organ(s) after death. However, your organ(s) can only be used if you have specifically authorized donation. Your family cannot authorize donation of your organs after your death. This type of consenting gives more weight to your autonomy but is associated with lower organ availability.

_________Donor-or-surrogate Informed Consent: You don’t have to decide whether you wish to donate (or not to donate) your organ(s) after death. However, your organ(s) can only be used if you have specifically authorized donation, or in case there is no directive from you, if your immediate family authorizes the donation of your organ(s) on your behalf.

_________Mandated Choice: You are required to decide whether you wish to donate (or not to donate) your organ(s) after death. Your decision should be declared and documented upon issuance of some official documents such as a national ID, driver’s license, or passport. Your family cannot authorize organ donation after your death. This type of consenting gives major weight to your autonomy but forces you to decide.

________Presumed Consent: Hospitals can procure organ(s) from you unless you have formally registered your objection to donation. If no objection is registered, it is assumed that you want to donate your organs. Neither you nor your family’s consent is required. This type of consent gives more weight to benefiting other people and is associated with higher organ availability.

__________ Donor-only Informed Consent plus Incentive A

__________ Donor-only Informed Consent plus Incentive B

__________ Donor-or-surrogate Informed Consent plus Incentive A

__________ Donor-or-surrogate Informed Consent plus Incentive B

__________ Mandated choice plus Incentive A

__________ Mandated Choice plus Incentive B

**What you think is the best for Saudi Society**

Please rank the following consenting options according to what you think is best for the Saudi society in general (regardless of what you prefer for your self). There are five main options and two kinds of incentives that can be combined with three of them. The options add up to a total of 11. Use each number only once. Please read all the 11 options before you answer.

Go over the 11 options. Select the best one. Give it number one. Then go over the 10 options left. Select the best one of the 10 and give it number 2. Then go over the 9 options left, select the best one and give it number 3. Continue until you rank all the options, 1 for the best and 11 for the worst.

*Incentive A: If an individual indicates his/her willingness to donate organ(s) after death, the individual and his/her family will be prioritized for receiving organs from other donors.*

*Incentive B: If an individual registers his/her willingness to donate organ(s) after death, or his/her family authorizes the donation of his/her organ(s) after his/her death, a financial donation to the donor’s (or donor’s family) charity of choice will be given by a third party.*

_________No-organ Donation: There should be no cadaver organ donation at all.

_________Donor-only Informed Consent: An individual doesn’t have to decide whether he/she wishes to donate (or not to donate) his/her organ(s) after death. However, an organ can only be used if the individual has specifically authorized donation. The family of the deceased cannot authorize organ donation. This type of consenting gives more weight to individual autonomy but is associated with lower organ availability.

_________Donor-or-surrogate Informed Consent: An individual doesn’t have to decide whether he/she wishes to donate (or not to donate) his/her organ(s) after death. However, an organ can only be used if the individual has specifically authorized donation or, in case there is no directive from the deceased, if the immediate family authorizes the donation on his/her behalf.

_________Mandated Choice: All competent adults should be required to decide whether they wish to donate (or not to donate) their organs after death. This decision should be declared and documented upon issuance of some official documents such as a national ID, driver’s license, or passport. The family of the deceased cannot authorize organ donation on his/her behalf. This type of consenting gives major weight to individual autonomy but forces the individual to decide.

_________Presumed Consent: Hospitals can procure organ(s) from the deceased unless the deceased has formally registered his/her objection to donation. If no objection is registered, it is assumed that the deceased wanted to donate his/her organ(s). No individual or individual’s family consent is required. This type of consent gives more weight to benefiting other people.

__________ Donor-only Informed Consent plus Incentive A

__________ Donor-only Informed Consent plus Incentive B

__________ Donor-or-surrogate Informed Consent plus Incentive A

__________ Donor-or-surrogate Informed Consent plus Incentive B

__________ Mandated choice plus Incentive A

__________ Mandated Choice plus Incentive B
